# A Suggestion on the Origin of Some Cases of Pyorrhœa Alveolaris

**Published:** 1896-05

**Authors:** H. C. Matlack


### A Suggestion on the Origin of some Cases of Pyorrhœa Aveolaris.

BY DR. H. C. MATLACK.

Read before the Mississippi Valley Dental Society.

We sometimes find men who will hesitate to advance an opinion which has a direct medical bearing, thinking that to be dogmatic and self-assertive without due reason is illogical and unjustifiable—which presents the spread of some thought and the solution of some great question. The following will be presented for what it is worth. I will try to show that there are *some* cases of pyorrhœa alveolaris whose origin may be syphilitic.

On October 14, 1895, a lady 28 years of age presented herself for treatment on account of looseness of upper front teeth. The four upper incisors were found to be very loose, gums could be lifted from necks of teeth, pus exuding, and the general conditions present which caused a diagnosis of pyorrhœa alveolaris, although no calcareous deposit was found on these or any of the other teeth—pyorrhœa instruments being used to determine whether there was such a deposit or not. The lady has been a patient of mine for several years, so that any inclination to salivary calculus or other deposits due to carelessness in cleansing the teeth would have been noticed. About eighteen months ago this lady was married to a young man who was *supposed* to have recovered from a severe attack of syphilis. On questioning the patient, I found that in May, '95, she had a severe case of iritis, which, by the time she was referred to an occulist, had developed into choroiditis. Now, from a number of authors, we find that choroiditis is a frequent disease in all ages, and the most ordinary cause is syphilis, both acquired and hereditary. Upon consulting a number of writers, we find that we have syphilitic iritis, choroiditis, deafness, otorrhœa, or a flow from the ear, syphilitic headache, produced by the virus irritating the membranes of the brain and pericranium; syphilitic complaints in the nose, originating from the immediate application of the virus to the nostrils; syphilitic sore-throat; syphilitic swelling of the bones, where may be traced one of the causes of exostosis of the teeth. The following, under the head of "Syphilitic Toothache," taken from a work published as early as 1801, shows that even then some diseases of the teeth were attributed to syphilis.

"The syphilitic virus, in affecting the eyes, the mucous membrane of the nose and throat sometimes attack the gums, producing syphilitic toothache, which must be carefully distinguished from that produced by mercury or mercurial odontalgia." Showing that attention was called to the difference between ptyalism and some other affection of the gums, perhaps pyorrhœa. In order to substantiate the theory of syphilitic infection causing pyorrhœa alveolaris, we must find it affecting the *periosteum* as well as the bone itself. No portion of the body is exempt from the attacks of syphilis. Stanley has the following, which shows that there is such a thing as syphilitic periostitis: "Syphilis is a well-recognized cause of inflammation of the periosteum. Systems debilitated by mercury and other drugs are thus rendered particularly susceptible to the influence of cold and moisture, and inflammation of the periosteum of one, and oftentimes *several*, bones is of *common* occurrence. The reason of this is probably because the surfaces of the bones, superficially situated, are most exposed to external influences, that these are the usual seat of periostitis. Acute inflammation of the periosteum occasions *increase* of its *vascularity—thickening* and *softening* of its tissues—*loosening of its connection with the bone—serous* or *purulent effusions between it and the bone.*"

Although it appears from a clinical history and pathological changes that the diseased action is marked at the extremities of the long bones, where nutritive changes are the most active, it is not confined to the ends of the long bones, and does not manifest itself in the interior of those parts of the extremities of the bones which are not covered externally by the periosteum, which gives the conclusion that in the majority of cases the periosteum is the tissue *primarily* involved in the diseased action, the bone being implicated in consequence of the periosteal abnormalities frequently occurring either before or during the eruptive stages of primary syphilis. During these early stages, although the pericementum is affected at the time, the patient is unable to localize the pain, which may be similar to locating an aching tooth; but later on, when the eruptive period of the disease is developed, the gums become excessively tender on pressure. These conditions being present in the case cited caused a further consideration of the subject. By the kindness of Drs. Evans and Ravogli, the City Hospital was visited, and twenty-three syphilitic patients were examined, eleven having true pyorrhœa. Now, to counteract the thought that there were probably cases of ptyalism, the attending physicians stated that although a small amount mercury was used, no ptyalism had been produced in any case. The case referred to in the beginning was treated by the usual methods; three of the teeth responded readily to the treatment, but the upper right lateral was not cured the last time I saw the patient.

I had intended taking up another phase of the subject, that is, that some cases of pyorrhœa aveolaris may be of a tubercular origin; but, as this paper was intended to be a suggestion only, the further consideration of the *tubercular idea* will be postponed to some future date.

DISCUSSION.

PROF. W. H. KNIGHT: The essayist, Dr. Matlack, has presented an interesting subject, and one which shows the relations between medicine and dentistry, by demonstrating the need of constitutional treatment in addition to local.

Pyorrhœa alveolaris, although usually arising from local causes alone, is at times associated with and partly caused by constitutional disturbances. Some of the specific and wasting diseases, by lowering the vitality of the tissues, render them much more vulnerable to the injurious effects of local irritants, so that a slight local cause—which acting upon the healthy parts —in possession of their normal resistance, would be inadequate to cause mischief, can do so under depressed or diseased condition of the parts. The hydra-headed disease to which the essayist has referred, can in some, although in rare instances, be a factor in the origin of a pyorrhœa alveolaris. Most authorities, among whom may be mentioned Bumstead and Hutchinson, agree that a syphilitic periostitis may be primary, that is, it may develop independently of any affection of the neighboring soft parts. Although it is the rule for syphilis to attack that part of the periosteum which covers compact bone, it is not at all surprising that this disease, which assumes so many phases, will in some rare instances invade the periosteum of the alveoli, and thus establish a pathological condition in this membrane which will make it exceedingly susceptible to the baneful influences of slight local irritation.

A case, in instance, occurred in a young man who some years ago consulted me in regard to an extremely offensive ozena. For some months previous to this he had been under the care of his dentist, suffering with pyorrhœa alveolaris accompanied with loosening of several of his teeth. Investigation of this patient revealed him to be a victim of congenital syphilis. He was placed upon constitutional treatment for the disease. His improvement was steady and continuous, the pyorrhœa alveolaris, which had resisted prolonged local treatment, disappeared, and the teeth became gradually tightened.

Dr. Fletcher: It is a matter of much interest to me to hear this paper and to know that some one has taken up the subject. As to the effects of syphilis as an exciting cause in cases of pyorrhœa alveolaris, I know very little, but can easily imagine it being one of the causes of this disease. I believe that pyorrhœa, as usually talked of, is considered to arise from a single cause, but, to me, there may be many causes, syphilis being one of these. Another cause mentioned by the essayist takes form in my mind as being one which may be frequent, and that is tuberculosis.

My hypothesis about the matter would be after this manner: We know that the bacilli of tuberculosis (when the patient has become affected) have a tendency to form colónies in various tissues of the body, and especially the endeothelial and epithelial membranes, such as the joints, the lungs and the mucous membrane of the air passages, and often in the skin, when it is called lupus; these tubercles are first invisible, when they are called granular, when a little larger they are called crude and then miliary tubercle; the last is so-called from its being about the size of a millet seed, and they may ultimately be much larger.

When the miliary size is reached, the tubercle may become caseous and then begin to break down in the center, not forming true pus, but a watery, milky fluid, just such a discharge as is often found in these pockets about the teeth. No doubt many of you have seen just this character of discharge from them. Now, the physical character of the two discharges being identical leads me to think that possibly many cases of pyorrhœa may be of tubercular origin. This could be determined by microscopical examination of the discharge, and I trust that the essayist, or some one else, may take up the subject from this standpoint and give us some facts and statistics relating to the tubercular origin, as well as the syphilitic or other cause of the disease.

